# Expanding the role of the oncology nurse

**DOI:** 10.2349/biij.4.3.e34

**Published:** 2008-07-01

**Authors:** A Quinn

**Affiliations:** University of Pittsburgh Cancer Centers, Radiation Oncology, Pittsburgh, Pennsylvania, United States

**Keywords:** Nursing, cancer, oncology

## Abstract

Oncology nursing continues to evolve in response to advances in cancer treatment, information and biotechnology. As new scientific and technological discoveries are integrated into cancer care, oncology nurses need to play a key role in the management of this patient population. The role of the oncology nurse has expanded significantly and can differ greatly across cultures. Sophisticated treatments and the growth of targeted therapies will create the challenge of ensuring that all nurses working in this arena are well-educated, independent thinkers. Thus the future success of oncology nurses will focus on enhancement of nursing practice through advanced education. The increased globalisation of healthcare offers exciting opportunities to accomplish this goal by allowing for collaborative relationships among oncology nurses across the globe.

## THE ROLE OF ONCOLOGY NURSES

Historically, nurses have played a special role in the care of patients with cancer, a role that was especially significant in those few institutions devoted exclusively to cancer care before the United States of America National Cancer Act of 1971. However, the recognition of cancer as a major American health problem and the subsequent expanded research and treatment program against cancer, which has occurred during the past quarter-century, has been a catalyst for the development of oncology nursing as a separate specialty. At first many oncology nurses worked as nurses and data managers for cancer research studies, but as the treatments in oncology became increasingly complex so did the need for a collaborative relationship between the nurse and physician in order to provide unique comprehensive patient care.

Today oncology nurses in the United States practise in a variety of settings, including acute-care hospitals, outpatient clinics, private oncologists’ offices, radiation therapy facilities, home healthcare agencies and community agencies. They may practise in surgical oncology, gynaecologic oncology, bone-marrow transplantation, radiation oncology, paediatric oncology or medical oncology. The majority are involved in direct patient care, with 35 percent working in a hospital/multi-hospital system, 40 percent in the outpatient/ambulatory care setting, 20 percent in radiation oncology, and 5 percent in hospice or home care [1]. The roles of the oncology nurse vary from the intensive care focus of bone marrow transplantation to the community focus of cancer screening, detection and prevention. Oncology nurses in the U.S. also tend to specialise in certain cancer types, working in areas such as breast cancer centers or lung cancer clinics.

Nurses working in cancer care focus on patient assessment, education, symptom management, and supportive care. In medical oncology they play an integral role in the administration of antineoplastic agents and are responsible for safe drug handling; evaluation of laboratory data; calculation of drug dosages on the basis of body surface area; insertion of intravenous lines or accessing central venous devices; continuous and time intensive monitoring to address potential adverse reactions or drug interactions; and screening patients for inclusion in available research trials or protocols [2]. In the radiation oncology arena the nurses need to have an understanding of radiobiology and radiation physics. They are also responsible for extensive symptom management, patient education and the submission process for clinical trials or research protocols.

As more complex treatment protocols are implemented, nurses working in oncology will need to expand their knowledge base on new drugs, new technologies, and biologic therapies. For example, The National Cancer Institute (NCI) announced in January 2006 that intraperitoneal (IP) combined with IV chemotherapy postoperatively was the preferred treatment method for advanced ovarian cancer. IP administration allows a high concentration of chemotherapy to come into direct contact with tumours and surrounding tissues and organs. The announcement stimulated the need for oncology nurses to become familiar with IP chemotherapy administration and patient management guidelines. These patients require constant monitoring of renal and cardiac function through laboratory values as well as intake and output to prevent fluid overload and electrolyte imbalances. The patients also need advanced nursing assessment to prevent any complications from the infusion.

Advances in molecular science have led to new biologic therapies for patients with cancer. These biological agents have created a challenge and require nurses to have a thorough understanding of their mechanism of actions and side effect profile. Patients may continue on these medications at home thus requiring the nurse to do a complete assessment of the knowledge level of the patient and/or caregivers regarding the preparation and administration of the medication at home, as well as management of possible side effects in the home setting.

In Asian countries the role of the oncology nurse continues to expand as cancer becomes a leading health concern. However, across Asia there is growing acknowledgement of the need to clarify the role of nurses in order to maximise their contribution to cancer care. Asia has many faces and is extraordinary in its diversity of cultures, habits, and healthcare systems. Oncology nurses in Asia function mainly in a caregiver role focusing on treatment delivery, education and symptom management. Specialisation is rarely seen. A study in 2005 by Gopal et al looked at information needs of women with newly-diagnosed breast cancer in Malaysia and the United Kingdom. Malaysian women in this study emphasised the importance of medical information on prognosis and spread of disease and the need for more education. Although nurses specialising in breast cancer are not features of the Malaysian healthcare system, the findings from this study support the view that specialised nurses may have a vital role to play [3].

Standards of practice and competencies for oncology nurses appear to be similar across continents [4]. Oncology nurses in an outpatient medical oncology clinic in Thailand, just as in the U.S., are responsible for starting their own intravenous lines, triaging patient phone calls, calculating absolute neutrophil counts, administering chemotherapy and reporting all relevant laboratory, pathology and imaging studies. In Thailand chemotherapy is generally mixed by the pharmacy except in smaller hospitals where the nurses are required to mix their own. Unlike the U.S., double-checking the dose of chemotherapy by calculating the body surface area (BSA) is the responsibility of the pharmacist. In radiation oncology the nurses once again have similar competencies to those nurses in the U.S. with a focus on symptom management and patient education.

Universally the oncology nurse has tremendous responsibility in educating the patient about his or her cancer treatment and often has better opportunities than any other member of the healthcare team to review the treatment plan. However, for some Asian countries the challenges of education extend well beyond diagnosis and treatment. In certain areas a diagnosis of cancer is taboo and rarely discussed within the family and never with outsiders. For example, breast cancer literature in Malay languages, even in the official Bahasa Melayu language, did not exist largely because of cultural mores regarding privacy about women's bodies, lack of education about the disease and the lack of public hospitals to prescreen women and provide early diagnosis as well as treatment options.

The use of alternative medicines is also a common practice in Asian countries and oncology nurses in these areas need to be familiar with the role such medicines play in cancer treatment. According to the World Health Organization (WHO), up to 80 percent of developing countries' populations use traditional medicines as their primary source of health care [5]. Those diagnosed with cancer in Southeast Asia will routinely find the local doctor's choice of treatments something many medical insurers consider unusual. Outside major cities in places like China, for instance, herbal treatments are used regularly . Although alternative therapies are becoming more common in the US, the oncology nurses in Asia must routinely educate patients regarding the use of such alternative treatments within the context of different cultural values. Furthermore, it is not unusual for a patient to be offered treatment with standard Western-style protocols with the addition of alternative therapy.

## NURSING WORKFORCE

In 2000, the number of new cancer cases in Asia almost exceeded the combined incidence of new cancer cases in North America, Europe and Australia combined, and is predicted to increase further [6]. WHO has projected that new cancer cases in Southeast Asia will rise from 1.3 million to 2.1 million between 2002 and 2020 - a dramatic 60 percent jump [7]. With the rising incidence and prevalence of cancer, the need for adequate nursing staff is becoming urgent.

One theme that rings true globally is the lack of nursing personnel (Table 1). A nursing shortage is an organisational challenge and has a major negative impact on healthcare. The failure to provide adequate levels of nursing staff has led to the undesirable trend of poor nursing services, as 2005 International Council of Nursing (ICN) makes clear. The average ratio in Europe is 10 times that in South East Asia, where the latter region also suffers from a poor distribution of nurses, with few nurses available in rural and remote areas [8]. The demand for nurses in many countries in Asia is so staggering that it has led to many nurses migrating due to better financial rewards in highly developed areas [9]. In the highest paid areas of the United States – for example, California – an average starting salary for a nurse ranges from $36,000- $45,000 a year, compared to nurses in Malaysia who have an average starting salary of $4,000 a year. The maximum salary in the U.S. for a nurse with more than 10 years of experience is around $58,000 while in Malaysia advanced practice nurses typically make around $10,000 a year. In China nurses in the smaller cities make as little as $200 a month. In the larger cities the nurses can make $1,000 a month, but these jobs are very limited.

**Table 1:** Number of nurses per 100,000 population.

Region	Nurses
Africa	17
Americas	212
South East Asia	45
Europe	327
Eastern Mediterranean	96
Western Pacific	157

According to the 2005 ICN Asia Workforce there is little difference in wages, benefits and working conditions between nurses in the private hospitals and those in the public hospitals. However, Malaysian nurses in private hospitals are paid more than nurses in public hospitals. This contrasts with Taiwan where the starting salary for nurses in public hospitals is higher than that offered by private hospitals. In Singapore, the basic pay in both sectors is similar [10].

Educational preparation also varies depending on the country. Thailand’s entry level into practice is at the baccalaureate level, but in other countries one can practise after completing a two-year diploma program. Most of the Asian countries require nurses to sit for licensing examinations in their national language, although this is a fairly new practice. In Thailand, the first national examination for nursing graduates was administered by the Nursing Council in 1998. Prior to this, graduation from an approved school of nursing or university was all that was required for licensing. The license is renewed every five years. In Taiwan approximately 25 percent of nurses have a baccalaureate degree [11]. In contrast, in 1905, the state of North Carolina required nurses who had successfully completed formal education programs to pass a licensing exam. According to the most recent survey from the Oncology Nursing Society approximately 48 percent of nurses in the U.S. hold a bachelor’s degree [1].

Advanced nursing education varies tremendously between the U.S. and Asian countries. In the U.S. the development of the advanced practice role began over 35 years ago in response to a shortage of primary care providers in rural areas. In 1989, nurse practitioner programs were required to be master’s granting programs or post-master’s degree programs. In contrast, in Asia the role regarding advanced practice nursing first emerged in Taiwan in 1994. Before that nurses in Taiwan were reluctant to work in an advanced practice capacity as it was considered illegal. Today advanced nursing practice in this country is booming but the standards of education have not been well-developed. The majority of the advanced practice nurses are either junior college graduates with four years of clinical experience or bachelor degree nurses with two years of clinical experience [12]. Short-term hospital-based continuing education courses are the only way of preparing advanced practice nurses in Taiwan.

## FUTURE OF ONCOLOGY NURSING

It has been estimated that there will be another 20 million new cancer patients worldwide in 2020 [13]. In the developing countries of Asia this poses a huge burden on an already taxed healthcare system. When the rise in cancer rates is coupled with concurrent therapies, targeted therapies and advanced treatment technology, the need for advanced practice nurses becomes extremely important. Enhancing the oncology nurse’s education is the main goal of the future and will allow nurses to have a greater contribution to cancer care in developing countries.

As stated previously, throughout Asia there is great diversity of educational preparation. Nurses are, at best, given a broad overview of cancer care in their basic educational programs, yet to practise in oncology, nurses must quickly learn the language of this discipline. Each type of cancer has a different etiology, pathophysiology, natural history and course of treatment. The number of chemotherapeutic agents and drug combinations, as well as targeted agents being added to treatment regimes is staggering. In radiation oncology, advances in technology have led to more defined treatment and a greater need for patient education.

A decade ago, an oncology nurse could become an expert in one treatment modality such as surgical, medical or radiation oncology. Now patients frequently receive concomitant and sequential therapies that require assessment and management skills for all three modalities. Nurses now have subspecialties such as breast care nurses, palliative care nurses, stem-cell transplant nurses and so on. In the larger cities of Asia, nurses may be familiar with newer technology, but more education is needed for nurses working in remote, less-developed areas of the country. As relevant healthcare systems are put in place to manage the rapidly increasing numbers of cancer diagnosed in this part of the world, there will be a greater need for education of oncology nurses who have never been exposed to managing skin rashes from targeted therapies or radiation therapy equipment. For instance, the WHO estimates that the Asia-Pacific region needs 4,000 radiotherapy machines to treat its patients, but has only 1,200 [14]. If an increase in radiotherapy centres is the future of cancer care in this area, then many more nurses will need to become experts in radiation oncology.

Although some countries require their nurses to have continuing education credits, this is not the standard for all countries. Programs focusing specifically on oncology are rare for nurses practising outside a major city. This leaves a lack of further education on newer treatment modalities.

Oncology nurses in Asia will also need to take an active role in developing prevention programs for cancer. The rapid rate of economic development in some Asian countries, along with the accompanying industrialisation and urbanisation, are contributing to an ever-increasing risk of common cancers. In Thailand 57 percent of boys begin smoking between the ages of 15 and 20, and unfortunately most countries in Asia have weak policies and programs for tobacco control [15].

Abundant evidence in the U.S. has demonstrated the benefits of the advanced nursing practice [16-18]. In the developing countries of Asia, advanced oncology nurses can also be instrumental in creating cancer prevention programs. For example, cervical cancer is the most common carcinomatous lesion in women in Thailand, accounting for 18.1 percent of all cancers found in Thai women [19]. In 2002, the Alliance for Cervical Cancer Prevention and the Thai Ministry of Public Health (MOPH) examined an innovative approach to cervical cancer prevention in Thailand [20]. Twelve nurses with advanced training used visual examination of the cervix with acetic acid (VIA) and cryotherapy to provide testing and treatment to women in a rural area of the country. Over 7 months, 5,999 women were tested for cervical cancer or pre-cancer with VIA. If they tested positive, they were given counseling and offered cryotherapy and further counseling regarding its benefits. The results of the project indicated the VIA and cryotherapy performed by advanced practice nurses was safe and feasible. Moreover it provided a cost-effective approach to providing cancer screening and treatment to women in the rural areas of Thailand where a more traditional approach to cancer prevention is low [21].

## CONCLUSION

For the oncology nurse, the learning curve is steep. The integration of targeted therapies into practice, advances in combined modality therapy and an increase in treatment delivery technology means that the oncology nurse must be well-educated to care for this population of patients. Oncology nurses must be able to think critically, analyse, reflect, problem-solve, and apply high-level knowledge that is evidence- and research-based to clinical interactions with patients who need their care. For some Asian countries, advanced practice nurses do not exist or they exist with a lack of role-clarity and educational preparation.

With cancer rates in Asia on the rise and an increase in complex treatments, there is a greater need for the advanced practice nurse. Advances in information technology can allow us to bring educational opportunities to nurses across the globe and establish a uniform process of educating and credentialing advanced practice nurses. The oncology nurse of the future will need to become comfortable and flexible with technology. Creating a global advanced oncology nursing curriculum can help to bridge the gap between oncology nurses across continents and enhance education of nurses working in the cancer setting in Asia.

## References

[1] Oncology Nursing Society (2008). Demographics report, as of April 20, 2008.

[2] Mick J (2008). Factors affecting the evolution of oncology nursing care. Clin J Oncol Nurs.

[3] Gopal RL, Beaver K, Barnett T (2005). A comparison of the information needs of women newly diagnosed with breast cancer in Malaysia and the United kingdom. Cancer Nurs.

[4] Anders R, Kunavikkikuk W (1999). Nursing in Thailand. Nursing and Health Sciences.

[5] World Health Organization WHO Traditional Medicine Strategy 2002-2005.

[6] Eaton L (2003). World cancer rates set to double by 2020. BMJ.

[7] World Health Organization (2005). Preventing chronic diseases: a vital investment [Online].

[8] International Council of Nursing (2006). Overview Paper 2006. Asia Workforce Forum.

[9] Pearson A (2004). The shortage of nurses: is it 'man'-made?. Int J Nurs Pract.

[10] (2005). International Council of Nursing Asia Workforce Forum 2005 [Online].

[11] Chen C (2001). The Current Issues in Advanced Practice Nursing in Taiwan [Online].

[12] Kamajian MF, Mitchell SA, Fruth RA (1999). Credentialing and privileging of advanced practice nurses. AACN Clin Issues.

[13] Lim GC (2002). Overview of cancer in Malaysia. Jpn J Clin Oncol.

[14] Bogda K, Brton M, Coventry B (2005). Cancer curriculum in the Asia-Pacific: Opportunities and challenges in the age of globalization. Asia-Pacific J Clin Oncol.

[15] Choe MK, Raymundo CM, The youth tobacco epidemic in Asia (2001). Honolulu, Hawaii: East-West Center.

[16] Ritz LJ, Nissen MJ, Swenson KK (2000). Effects of advanced nursing care on quality of life and cost outcomes of women diagnosed with breast cancer. Oncol Nurs Forum.

[17] Ray GL, Hardin S (1995). Advanced practice nursing: playing a vital role. Nurs Manage.

[18] McGee P, Castledine G, Brown R (1996). A survey of specialist and advanced nursing practice in England. Br J Nurs.

[19] IARCPress (2001). GLOBOCAN 2000: Cancer incidence, mortality, and prevalence worldwide. Version 1.0..

[20] Corneli A, Gaffikin L, Baldwin L (2003). A qualitative evaluation of the acceptability and feasibility of a single visit approach to cervical cancer prevention.

[21] Goldie SJ, Kuhn L, Denny L (2001). Policy analysis of cervical cancer screening strategies in low-resource settings: clinical benefits and cost-effectiveness. JAMA.

